# Increased Community-Associated *Clostridioides difficile* Infections in Quebec, Canada, 2008–2015^1^

**DOI:** 10.3201/eid2606.190233

**Published:** 2020-06

**Authors:** Veronica Zanichelli, Christophe Garenc, Jasmin Villeneuve, Danielle Moisan, Charles Frenette, Vivian Loo, Yves Longtin, Québec C.difficile Infection Surveillance Program (SPIN-CD)

**Affiliations:** Sir Mortimer B. Davis Jewish General Hospital, Montreal, Quebec, Canada (V. Zanichelli, Y. Longtin);; Centre de Recherche du CHU de Quebec, Quebec City (C. Garenc);; Institut National de Santé Publique du Québec, Quebec City, Quebec, Canada (C. Garenc, J. Villeneuve);; CSSS Rivière-du-Loup, Rivière-du-Loup, Québec, Canada (D. Moisan);; McGill University Health Centre, Montreal (C. Frenette, V. Loo, Y. Longtin)

**Keywords:** *Clostridioides difficile*, community-associated infection, CDI, bacteria, Quebec, Canada, enteric infections

## Abstract

The annual incidence rate of community-associated *Clostridioides difficile* infections in Quebec, Canada, has increased by 33.3%, from 0.51 (2008) to 0.68 (2015) cases/100,000 population, while incidence of healthcare-associated cases remained relatively stable. Possible causes include increased disease severity, increased antimicrobial drug use, emergence of virulent strains, and heightened physician awareness.

*Clostridioides difficile* infections (CDIs) are commonly acquired in healthcare settings (*1*). In 2003, an outbreak of CDI in the province of Quebec, Canada (population, 8.2 million), required implementation of mitigation strategies and prompted introduction of a surveillance program (*2*,*3*). Afterward, incidence of healthcare-associated CDIs (HA-CDIs) in the province decreased from 13.7 cases/10,000 patient-days in 2004–2005 to 6.9/10,000 patient-days in 2014–2015. Although CDIs afflict mainly hospitalized patients, recent studies report increased incidence of community-associated CDIs (CA-CDIs) (*4*–*6*). Whereas most of the focus in North America has been on HA-CDI, we describe and compare long-term trends in incidence rates for HA-CDI and CA-CDI in Quebec.

## The Study

To evaluate HA-CDI and CA-CDI trends in Quebec, we performed a quasi-experimental study. We used prospectively collected data from the Quebec Health Ministry *C. difficile* Infection Surveillance Program (SPIN-CD), a mandatory surveillance program introduced in 2004 (*7*). As of 2018, all 90 acute-care hospitals with >1,000 admissions annually participate in SPIN-CD. These hospitals, which represent 97% of all admissions in the province, use a centralized web portal to report HA-CDI incidence density, computed as the aggregate number of cases divided by the aggregate number of patient-days per 4-week period. CA-CDI incidence rates are expressed as the number of CA-CDIs/100,000 population as reported by the Quebec Institute of Statistics (*8*).

CDI is defined as diarrhea (>3 unformed stools in <24 hours with symptoms lasting >24 hours) with no other etiology and either a positive toxigenic *C. difficile* test result or evidence of pseudomembranes during histopathologic or colonoscopic examination (*9*). A case is considered to be HA-CDI if symptoms appear >72 hours after admission or <4 weeks after discharge. A CA-CDI case is defined as illness in a hospitalized patient for whom symptoms developed within 72 hours of admission and who had not been hospitalized or received ambulatory care in the previous 4 weeks. Nonhospitalized CA-CDI case-patients and recurrences (i.e., new CDI episodes within 8 weeks of a previous episode) are not reportable to SPIN-CD. Definitions did not change during the study period. The type of laboratory assay used to detect *C. difficile* was at the discretion of each center (*10*).

To focus the analysis on the postepidemic period (April 2008–March 2015), we excluded data from the epidemic period (August 2004–March 2008). We used Poisson regression models on time series data, including trend and periodic seasonal terms to calculate incidence rate ratios (IRRs) with 95% CIs and to assess trends in CA-CDIs and HA-CDIs. We also used interrupted time series analysis with segmented regression because of a change in the level and trend in HA-CDI incidence rate from 2011 on (*11*). We compared the change in trends in HA-CDI and CA-CDI incidence by using Z-tests of the difference of the natural logarithm of incidence rates. We used SAS software version 9.3 (https://www.sas.com) for analyses and considered p<0.05 to be significant.

**Figure 1:**
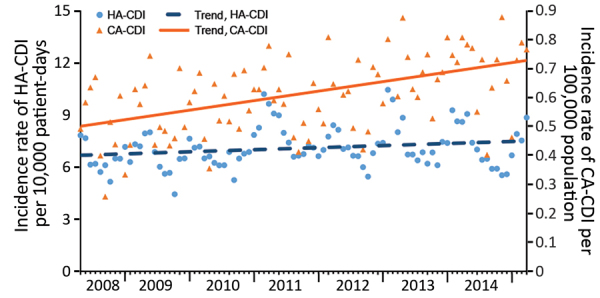
Incidence density of HA-CDIs and CA-CDIs per 4-week period, according to standardized surveillance definitions, Quebec, Canada, April 2008–March 2015. CDI, *Clostridioides difficile* infection; CA-CDI, community-associated CDI; HA-CDI, healthcare-associated CDI.

During the study period, a total of 28,854 cases of HA-CDI (84.5%) and CA-CDI (15.5%) were reported. The annual number of CA-CDI cases and the proportion of CDI cases that were reported as CA-CDI increased gradually from 510 (13.6% of HA-CDI) to 729 (17.8% of CA-CDI) cases (Table 1). Furthermore, the CA-CDI incidence rate increased ≈6.5% annually and overall significantly by 33.3%, from 0.51 to 0.68 cases/100,000 population (IRR per 4-week period 1.005, 95% CI 1.004–1.006; p<0.0001) (Figure 1). By contrast, the incidence of HA-CDI did not change significantly (IRR per 4-week period 1.000, 95% CI 0.999–1.000; p = 0.23). Accordingly, incidence rate trends for CA-CDI differed significantly from trends for HA-CDI (IRR 1.005, 95% CI 1.004–1.006; p<0.0001).

**Table 1:** Annual incidence rate of community-associated and healthcare-associated *Clostridioides difficile* infections in the province of Quebec, Canada, 2008–2015

Years	Community-associated cases					Healthcare- associated cases		
	No. cases	Mean annual population	Rate/100,000 population	% Total cases		No. cases	No. patient-days	Rate/10,000 patient-days
2008–2009	510	7,764,759	0.51	13.6		3,244	4,915,666	6.60
2009–2010	568	7,846,295	0.56	15.1		3,206	4,855,739	6.60
2010–2011	619	7,930,943	0.60	14.9		3,544	4,898,891	7.23
2011–2012	605	8,009,614	0.58	14.1		3,697	4,927,050	7.50
2012–2013	697	8,084,741	0.66	15.9		3,695	4,941,796	7.48
2013–2014	753	8,150,131	0.71	17.2		3,615	4,880,472	7.41
2014–2015	729	8,209,599	0.68	17.8		3,372	4,843,433	6.96

**Figure 2:**
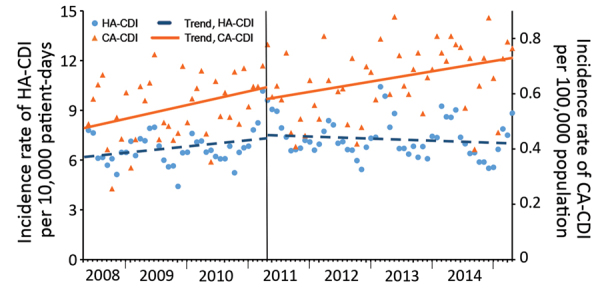
Trends in incidence of HA-CDIs and CA-CDIs analyzed by using linear segmented regression (inflection point of HA-CDI in April 2011) per 4-week period, according to standardized surveillance definitions, Quebec, Canada, April 2008–March 2015. CDI, *Clostridioides difficile* infection; CA-CDI, community-associated CDI; HA-CDI, healthcare-associated CDI.

An inflection point in HA-CDI incidence in April 2011 was demonstrated by a significant decreasing change in trend (IRR 0.996, 95% CI 0.994–0.998; p = 0.001). By contrast, no concomitant change occurred in the trend of CA-CDI at the inflection point (Figure 2, Table 2).

**Table 2:** Segmented regression of HA-CDI and CA-CDI in the province of Quebec, Canada, 2008–2015*

Rate	2008–2009 to 2010–2011			2011–2012 to 2014–2015			
	Overall trend before the breakpoint, IRR (95% CI)	p value		Immediate change after the breakpoint, IRR (95% CI)	p value	Change in trend after the breakpoint, IRR (95% CI)	p value
HA-CDI rate/10,000 patient-days	1.003 (1.001–1.005)	0.001		0.998 (0.945–1.053)	0.93	0.996 (0.994–0.998)	0.001
CA-CDI rate/100,000 population	1.007 (1.003–1.012)	0.002		0.95 (0.841–1.074)	0.41	0.997 (0.992–1.002)	0.30
Group difference	1.002 (0.997–1.007)	0.35		0.971 (0.851–1.107)	0.66	1.002 (0.996–1.008)	0.53

*Breakpoints were identified in April 2011. CA, community-acquired; HA, hospital-acquired; CDI, *Clostridioides difficile* infection; IRR, incidence rate ratio (calculated per 4-week period).

## Conclusions

Although the incidence of HA-CDI has been decreasing in Canada since 2009 (*12*), our study suggests possible emergence of CA-CDI in the province of Quebec because the number, incidence, and proportion of reported cases have been steadily increasing since 2008. This increased incidence contrasts markedly with the overall decreasing trend of HA-CDI incidence after April 2011.

Emergence of CA-CDI has been reported in other countries (*4*–*6*,*13*). A 2008–2013 study in Finland reported an increase in probable CA-CDI cases at an annual rate of 4.3% compared with a concomitant decrease in HA-CDI cases at an annual rate of 8.1% (*4*). In the United Kingdom, an analysis of hospital administrative data detected an increase in the proportion of CDI cases that were community acquired, from 7% in 1998 to 13% in 2010, while the overall incidence of CDI cases fell to less than half of peak rate (*13*). The US Veterans Healthcare Administration reported similar findings of an increased proportion of CDI cases that were community-associated (from 8.3% in 2003 to 26.7% in 2014) (*5*). Electronic patient records analysis in Hong Kong identified a 3-fold increase in the incidence of CA-CDI cases, from 0.86/100,000 population in 2006 to 2.96/100,000 population in 2014 (*6*). These reports suggest that CA-CDI may be increasing worldwide; however, because the studies relied on retrospective extraction of data that were not specifically collected for CDI surveillance, they may be susceptible to misclassification and reporting biases (*14*).

The factors underlying this apparent increase in CA-CDI incidence are unclear. However, we hypothesize that this rise may result from any combination of the following: increased disease severity, leading to a greater proportion of case-patients being hospitalized; increased use of antimicrobial drugs or proton-pump inhibitors in the community; emergence of community-specific novel virulent *C. difficile* strains; and heightened awareness by physicians to consider the diagnosis of CDI.

Our study has many strengths. We used prospectively collected data from a well-established surveillance program enrolling virtually all persons admitted to acute-care hospitals in the province, thereby limiting selection bias, and we used standardized case definitions to avoid misclassification issues. However, our study also has several limitations. Only persons hospitalized with CA-CDI were reported to the surveillance program; therefore, milder cases were not captured. Thus, the actual incidence of CA-CDI may be underestimated. Because no clinical data regarding patients in whom CDI develops were collected, we cannot characterize patients or investigate potential changes in the affected population. We could not investigate the effect of diagnostic assay modifications on the observed change in trend because this information was not collected. Nucleic acid amplification tests are more sensitive than enzyme immunoassays for detecting toxigenic *C. difficile*; thus, increased use of these tests could lead to increased CA-CDI incidence rates. However, use of a more sensitive assay would be expected to affect CA-CDI and HA-CDI incidence similarly, whereas these trends are clearly divergent.

In conclusion, CA-CDI incidence in the province of Quebec increased significantly during 2008–2015 despite an overall decrease in HA-CDI incidence. This divergence in trends suggests a need to devote more attention to CA-CDI. 
